# Preparation of Particle-Reinforced Resin Using Highly Functional ZnO Particle Filler Driven by Supramolecular Interactions

**DOI:** 10.3390/ma18132986

**Published:** 2025-06-24

**Authors:** Haruka Nakagawa, Kohei Iritani

**Affiliations:** 1 School of Engineering, Tokyo University of Technology, 1404-1, Katakuramachi, Hachioji 192-0982, Tokyo, Japan; d512500321@edu.teu.ac.jp; 2 Research Center for Advanced Lignin-Based Materials, Tokyo University of Technology, 1404-1, Katakuramachi, Hachioji 192-0982, Tokyo, Japan

**Keywords:** zinc oxide, bioplastics, surface modification, nanoparticles, hydrothermal synthesis, composites

## Abstract

The surface modification of zinc oxide nanoparticles (ZnONPs) with organic compounds has been shown to improve their dispersibility. In this study, to develop a highly functional material, ZnONP modified with 6-amino-1-hexanol bearing both amino and hydroxyl functional groups was synthesized. Scanning electron microscopy–energy dispersive X-ray spectroscopy (SEM-EDS) and X-ray photoelectron spectroscopy (XPS) analyses confirmed that functionalized ZnONP was successfully obtained by a hydrothermal synthetic method. The mechanical properties of composite films of polylactic acid (PLA) reinforced with the functionalized ZnONP were then evaluated. The composite containing functionalized ZnONP exhibited a higher maximum stress than that containing unmodified ZnONP. These ZnONP/polymer composites therefore show promise as novel high-performance materials.

## 1. Introduction

Metal oxides [1,2,3,4], such as ZnO, TiO_2_, and NiO, have a wide range of applications in electronic devices, energy-storage systems, and structural materials. Among them, ZnO has been used in everyday products and biomedical applications because of its UV absorption [5,6], antibacterial properties [7], biocompatibility [8,9], and affordability. To maximize its potential, ZnO nanoparticles (ZnONPs) have attracted intense interest for use in cosmetics, industrial films, and paints due to their UV-shielding ability [10,11,12,13,14,15]. Surface modification of ZnONP has been employed to prevent aggregation of fine particles and stabilize the suspension [7,11,16,17]. Moreover, functionalizing the ZnONP surface has enabled the creation of high-value-added materials. For example, Kumar and co-workers demonstrated control of oxidative stress in bacteria using ZnONP with surface-adsorbed isopropylamine, and a material for a biosensor was fabricated by chemically modifying ZnONP with triethanolamine to improve stability [18]. In addition, ZnONP has been used as a filler for a particle-reinforcing material. For example, Bao and Liu and co-workers reported an improvement in the dispersibility and antibacterial properties of polymer materials by physically dispersing ZnONP into PLA or PVA matrices [19]. Although high-performance ZnONPs have been produced through surface functionalization, their practical applications remain limited because they often respond selectively to a specific substrate.

Numerous synthetic methods for ZnONP have been developed [20,21,22,23,24,25,26,27,28,29,30,31,32,33,34,35,36,37,38,39,40]. For example, commonly used methods are sol–gel [20,21], hydrothermal [22,23,24], wet-chemical [25,26], and spray-pyrolysis [27,28] techniques. The hydrothermal method is particularly attractive because it is sustainable, operates at relatively low temperatures, and requires short processing times. Using this approach, chemical modification of ZnONP with hexanol has also been reported [24]. X-ray photoelectron spectroscopy (XPS) analyses are commonly used to evaluate surface modifications of ZnONP for interfacial bonding characteristics in metal oxide/carbon nanocomposites [41,42,43,44]. Nevertheless, comprehensive studies on creating high-value-added ZnONPs through surface functionalization are still scarce.

**Figure 1:**
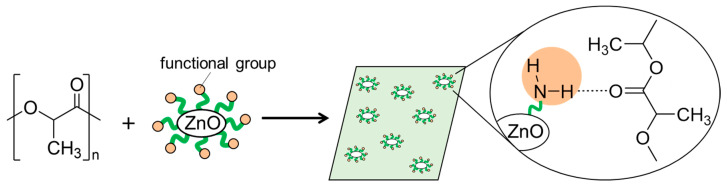
Schematic illustration for fabrication of composite of PLA with functionalized ZnONP; hydrogen-bond formation between amino group on ZnONP and carbonyl group attached with PLA.

In this work, we synthesized surface-functionalized ZnONP by the hydrothermal method and explored its use as a reinforcing filler in composite materials. Our goal was to obtain high-strength polymer materials through interactions between the polymer matrix and the functionalized ZnONP. We chose 6-amino-1-hexanol (6AH) as the modifier because its amino group would donate hydrogen bonds, and we selected polylactic acid (PLA) as the polymer matrix because its carbonyl oxygen atom would accept hydrogen bonds (Figure 1). PLA is a biodegradable polymer and therefore offers potential for eco-friendly materials. Because supramolecular interactions are highly versatile, introducing modifiers with diverse chemical structures should open the way to a wide variety of functional composites.

## 2. Materials and Methods

### 2.1. Reagents

Reagents used for the experiments were purchased from Fujifilm Wako Pure Chemical Industries, Ltd. (Osaka, Japan), and used without further purification.

### 2.2. Syntheses of Surface-Modified ZnONPs

Syntheses of ZnONPs were conducted according to the previously reported hydrothermal method [24], examining the temperature and time conditions due to the addition of 6AH. Zn(NO_3_)_2_∙6H_2_O (2.97 g, 10.0 mol) and KOH (1.12 g, 20.0 mol) were added in an autoclave. In the case of surface modifications, 6AH (6.23 g, 53.2 mmol) or 1-hexanol (6.67 mL, 53.2 mmol) was also added as a modifier. The autoclave was kept in an oven at a constant temperature (80 or 120 °C) for a fixed duration (1 or 24 h); subsequently, the product was collected by centrifugation (3500 rpm for 10 min) and washed with water (for the sample without the modifier) or ethanol and water (for that with the modifier).

### 2.3. Structural Analysis of ZnONP Samples

X-ray diffraction (XRD) measurements were carried out with a SmartLab 3 fully automated multipurpose diffractometer (Rigaku Corp., Tokyo, Japan). The wavelength used for the measurement was CuKα radiation. The crystallite size was averaged at three representative peaks observed within 2*θ* from 30° to 40°. The average crystallite size *D* was estimated using the Scherrer equation $D=\frac{K\lambda }{\beta cos\theta }$, where *K* is a dimensionless Scherrer’s constant, where a value of 0.9 is used as an approximation; *λ* is the X-ray wavelength, where a value of 0.15418 nm for CuKα is used; *β* and *θ* are full width at half maximum and Bragg angle, respectively, in radians. The *D* values were estimated as averages at three characteristic peaks of *θ* at 31.78, 34.37, and 36.25.

### 2.4. Evaluations of Surface Modification

Scanning electron microscopy (SEM) coupled with energy-dispersive X-ray spectroscopy (EDS) was performed on a JSM-IT200 (Keyence Corp., Osaka, Japan). In the case of SEM measurements for organic samples, each sample was Au ion coated by using an IB-2 ion coater (Eiko Corporation, Tokyo, Japan) for charge prevention. For the observation of ZnONP, the sample was prepared by attaching carbon tape to a brass sample stage and mounting the sample on it. For the observation of film, the sample was prepared by attaching carbon tape to the brass sample stage for cross-sectional observation and fixing the film. ZnONPs were analyzed by SEM-EDS using a secondary electron detector (Keyence Corp., Osaka, Japan) at an accelerating voltage of 20.0 kV, at a working distance (WD) of 10.5 mm under high vacuum conditions. Cross-sectional SEM-EDS analysis of the film was performed using a backscattered electron detector (Keyence Corp., Osaka, Japan). at an accelerating voltage of 15.0 kV, at a WD of 10.4 mm under low vacuum conditions. X-ray photoelectron spectroscopy (XPS) data were collected on a JPS-9200 photoelectron spectrometer (JEOL Ltd., Tokyo, Japan). XPS samples were prepared by attaching a paste of In to an Al plate cut to a 50 mm × 50 mm size and then embedding ZnONP samples on top of the paste. The XPS spectrum was measured using monochromatic X-rays (Al). Before XPS measurements, each sample was Au ion coated by using an IB-2 ion coater (Eiko Corporation, Tokyo, Japan). The binding energy scale was calibrated with reference to the Au 4f_7/2_ at 84.0 eV [45]. XPS measurements were performed using Al Kα radiation (*hν* = 1486.6 eV) under an accelerating voltage of 10 kV and a discharge current of 10 mA. The energy resolution and pass energy were set to 0.5 eV and 300 eV, respectively, for survey scans, and 0.3 eV and 100 eV, respectively, for O 1s and Zn 2p. Core-level spectra of O 1s and Zn 2p were analyzed after background subtraction using a Shirley method. Peak fitting was carried out using a mixed Gaussian–Lorentzian function, in which the ratio of these functions was also fitted.

### 2.5. Preparations of PLA Composite Films

ZnONP/PLA composite films were prepared by melting PLA on a hot plate at 220 °C and mechanically mixing ZnONPs of 0, 0.5, 1, 2, and 5 wt%. Subsequently, films were prepared by heating at 200 °C with pressure under 10 MPa. Figure S1 shows the appearance of the films. UV-vis transition spectra were measured with a UV-2600/2700 UV-vis spectrophotometer (Shimadzu Corp., Kyoto, Japan).

### 2.6. Tensile Testing of Films

Tensile tests were conducted on an EZ-SX tester (EZ Test series, Shimadzu Corp., Kyoto, Japan). To fit into the tensile strength tester, the films were cut to 5 mm × 50 mm using a cutter. The Young’s modulus (*E*) of the film was determined from the equation E= (W/A)/(X/L), where *W* is the applied load measured during the tensile test, *A* is the cross-sectional area of the sample, *X* is the elongation, and *L* is the initial gauge length. The load (*W*) and elongation (*X*) were obtained from tensile test data. The cross-sectional area (*A*) was calculated by multiplying the film width (5 mm) with its thickness, measured using a film thickness meter. The gauge length (*L*) was set to 10 mm for all samples. Stress and strain were defined as *W*/*A* and *X*/*L*, respectively, and the stress–strain curve was constructed from the tensile test results. The values of the mechanical properties of each film were the averages of 10 test pieces.

### 2.7. Evaluations of Films Based on FT-IR Measurements

FT-IR spectra were measured using IR Affiniry-1S (Shimadzu Co., Ltd., Kyoto, Japan) by an ATR method. The dried film was placed on a measuring plate at a room temperature under atmosphere and was sandwiched with a fixture to obtain spectra. The range was set to 400–4000 cm^−1^ with 256 scans accumulated. The Happ-Genzel function was used as the apodization function. XRD patterns of the films were measured using the same method described in Section 2.3. Peak fitting was carried out using a mixed Gaussian–Lorentzian function, in which the ratio of these functions was also fitted. Differential scanning calorimetry (DSC) analyses were conducted using a Thermo plus EVO2 DSC8231 (Rigaku Corp., Tokyo, Japan) over a temperature range of 40–200 °C with approximately 10 mg of each sample. Measurements were carried out under dynamic heating conditions at a fixed rate of 5 °C/min. Key thermal transitions, including the glass transition temperature (*T*_g_), cold crystallization temperature (*T*_cc_), and melting temperature (*T*_m_), were evaluated from the heating curves of the second heating cycle.

### 2.8. Water Repellency Test for Films

Static contact angles were measured with a Drop Master system (Kyowa Interface Science Co., Ltd., Saitama, Japan). All pictures were taken within 10 s after dropping water on the film surface. The values of the contact angles of each film were the averages of 5 test pieces.

### 2.9. Analyses for Particle Distribution in the Composite Film

Image analysis was performed by MATLAB (24.2.0.2871072 (R2024b); updated on 7 February 2025). Five images were analyzed for each sample. The binarized images were obtained using the following procedure: the original image was first converted to grayscale to transform color information into luminance values; a Gaussian filter (*σ* = 2) was then applied to suppress noise; adaptive thresholding, which determines local thresholds based on the neighborhood intensity distribution, was used to robustly extract target regions under varying illumination conditions; morphological erosion was subsequently applied to remove small artifacts and protrusions; finally, a hole-filling process was performed to close internal cavities within the segmented regions.

## 3. Results and Discussion

### 3.1. Structural Analyses of Products

**Figure 2:**
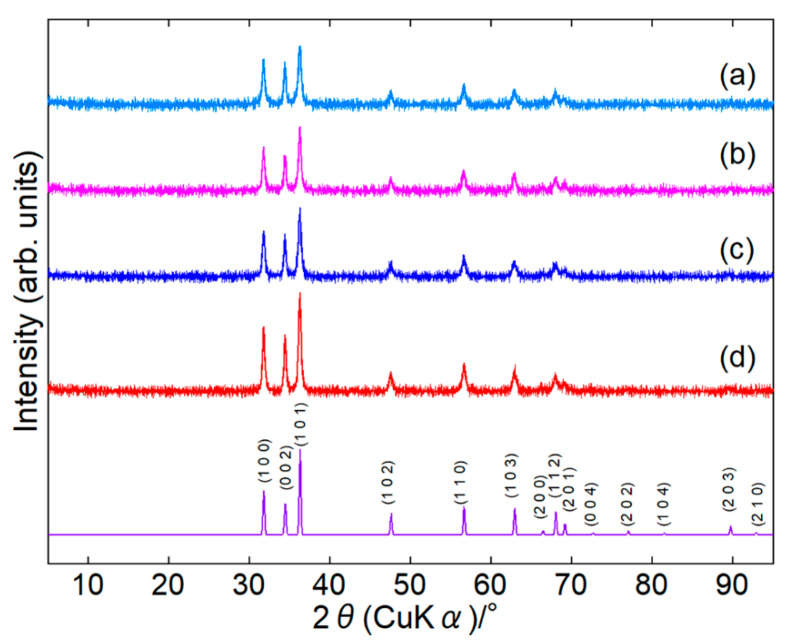
XRD patterns of ZnONP-80-1 (a), ZnONP-120-1 (b), ZnONP-80-24 (c), and ZnONP-120-24 (d).

Surface-modified ZnONPs were synthesized under four conditions: 80 or 120 °C for 1 or 24 h. In the paper, we represent the sample as ZnONP-temp.-time, for example, as ZnONP-80-24. XRD patterns confirmed that every sample crystallized as single-phase hexagonal wurtzite-type ZnO [46] (Figure 2 and Figure S2). In Table S1, their crystallite sizes are summarized.

### 3.2. Evaluations of Surface Modification

**Figure 3:**
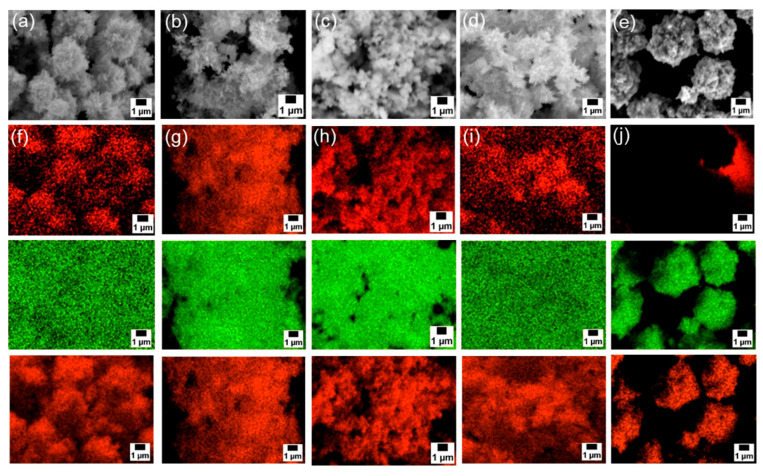
(**a**–**e**) SEM images and (**f**–**j**) EDS mappings (top: carbon, middle: zinc, bottom: oxygen) for modified ZnONP-80-1 (**a**,**f**), ZnONP-120-1 (**b**,**g**), ZnONP-80-24 (**c**,**h**), ZnONP-120-24 (**d**,**i**), and unmodified ZnONP-120-1 (**e**,**j**).

**Figure 4:**
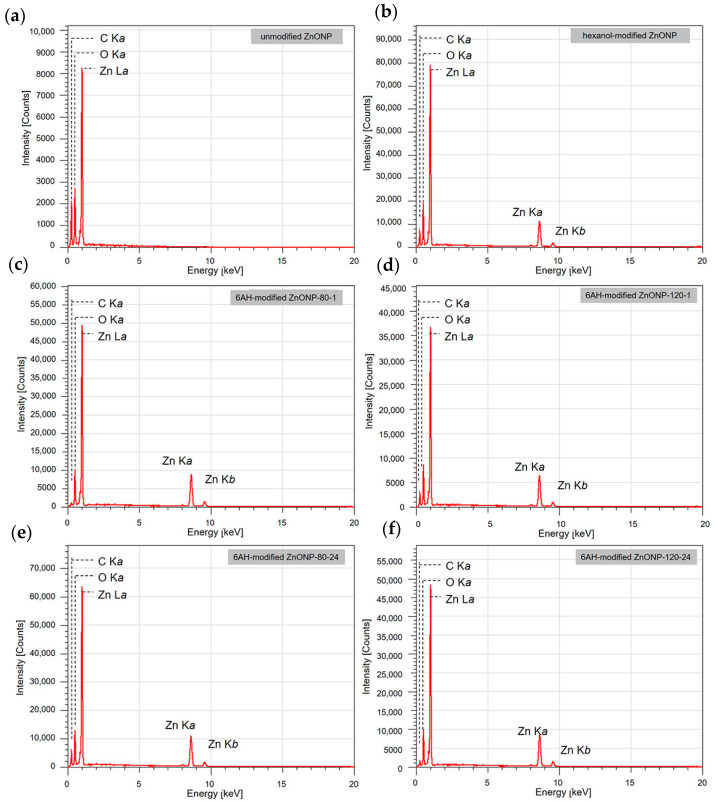
EDS spectra for unmodified ZnONP (**a**), 1-hexanol-modified ZnONP (**b**), 6AH-modified ZnONP-80-1 (**c**), ZnONP-120-1 (**d**), ZnONP-80-24 (**e**), ZnONP-120-24 (**f**).

Next, SEM-EDS analyses were carried out to examine particle morphology and elemental distribution. Figure 3a–e and Figure S3a presents SEM images; Figure 3f–j and Figure S3b–d show the corresponding EDS mapping for carbon, zinc, and oxygen; and Figure 4 exhibits EDS spectra of each sample. Although fine primary particles were present in all samples, they formed micrometer-scale agglomerates, likely during drying. In all cases, zinc and oxygen signals were detected. On the other hand, carbon signals appeared only in the samples modified with 6AH, while unmodified ZnO showed no surface carbon. Because the modifier was the only carbon source, these results confirm successful surface modification under all conditions for the 6AH and 1-hexanol modification.

**Figure 5:**
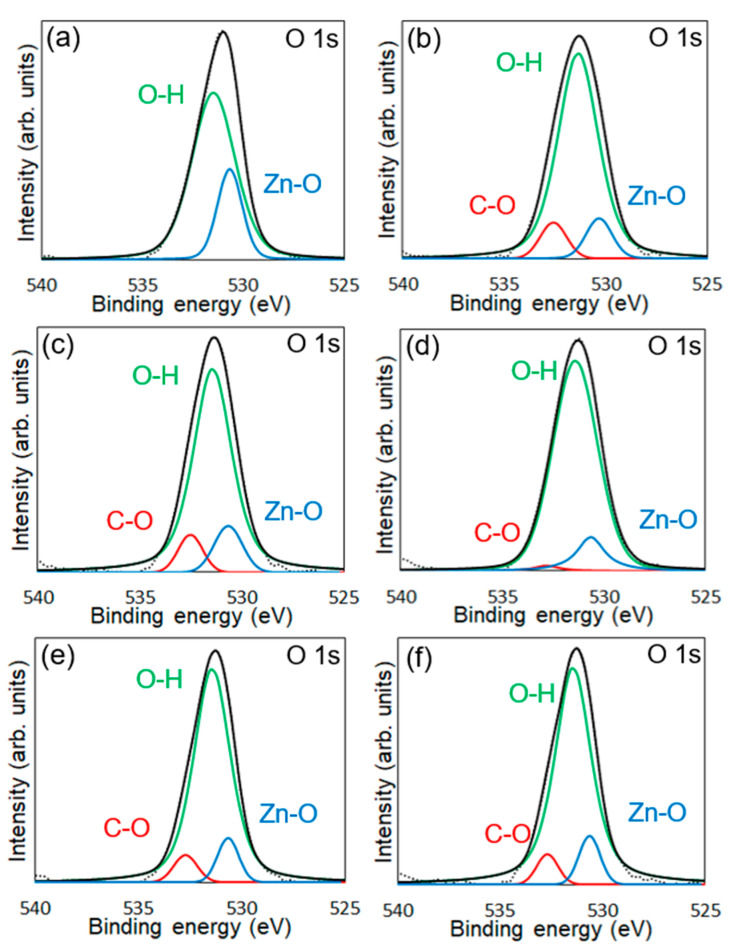
XPS spectra of the O 1s region for unmodified ZnONP (**a**), 1-hexanol-modified ZnONP (**b**), 6AH-modified ZnONP-80-1 (**c**), ZnONP-120-1 (**d**), ZnONP-80-24 (**e**), and ZnONP-120-24 (**f**). The black dotted, black solid, red solid, green solid, and blue solid lines indicate measured spectra, fitting curve, components for carbon-bonded, surface hydroxyl, and bulk oxygen atoms, respectively.

**Figure 6:**
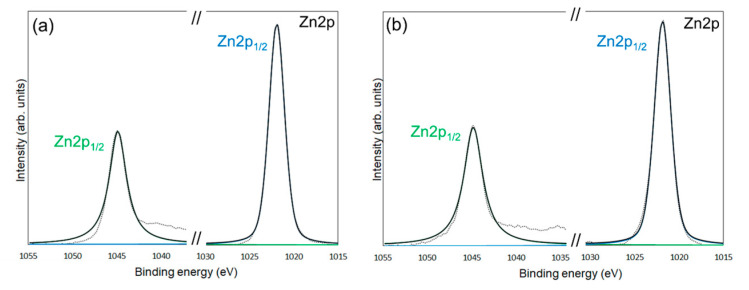

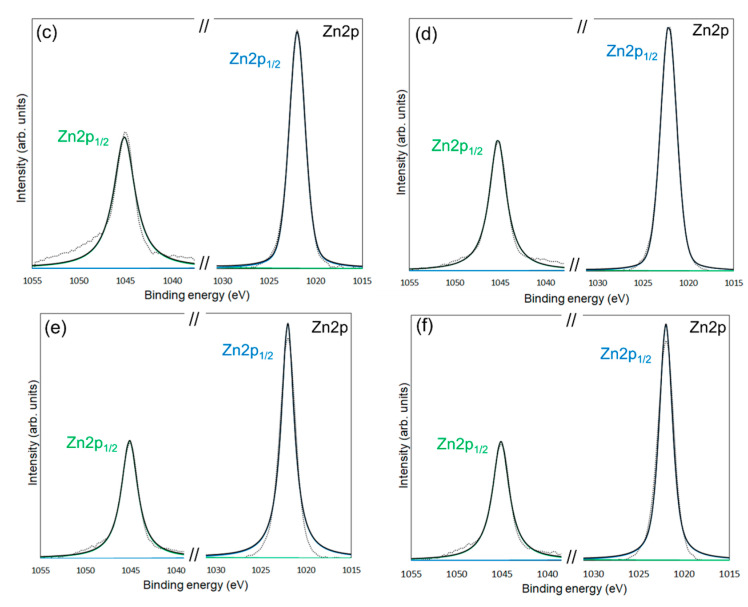
XPS spectra of the Zn 2p_1/2_ and Zn 2p_3/2_ for unmodified ZnONP (**a**), modified 1-hexanol-ZnONP-120-1 (**b**), 6AH-ZnONP-80-1 (**c**), 6AH-ZnONP-8-24 (**d**), 6AH-ZnONP-120-1 (**e**), 6AH-ZnONP-120-24 (**f**). The black dotted, black solid, green solid, and blue solid lines indicate measured spectra, fitting curve, components for Zn 2p_1/2_, and Zn 2p_3/2_, respectively.

**Figure 7:**
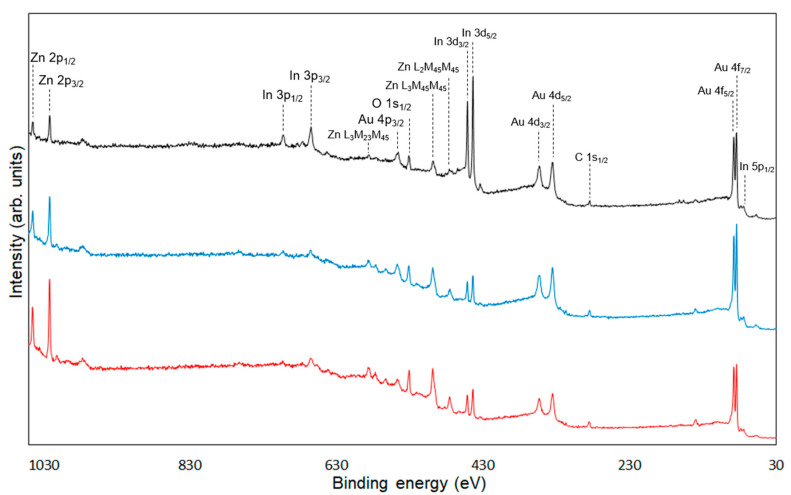
Survey spectra of unmodified ZnONP (black line), 1-hexanol-modified ZnONP (blue line), 6AH-modified ZnONP (red line).

XPS measurements were conducted to confirm the chemical modification of 6AH on the ZnONP surface. Figure 5, Figure 6 and Figure 7 present the XPS spectra of the O 1s and Zn 2p regions and survey spectra, respectively. In the survey spectra, peaks corresponding to In and Au used for the fixation of samples and the references of the binding energy, respectively, were observed as well as peaks attributed to ZnO. As shown in Figure 6, the Zn 2p_1/2_ and Zn 2p_3/2_ peak positions for all samples matched previously reported values [47]. Reported binding energies for bulk oxygen, surface hydroxyl oxygen, and carbon-bonded oxygen are 529.6, 531.2, and 532.7 eV, respectively [47]. Each O 1s spectrum was deconvoluted into three components by Gaussian–Lorentzian fitting using these reference values. The resulting binding energies and peak area ratios are summarized in Table 1. For unmodified ZnONP (Figure 5a), peaks at 530.7 and 531.5 eV correspond to bulk and surface oxygen, respectively, whereas the component attributable to carbon-bonded oxygen was too weak to appear as a distinct peak. In contrast, all 6AH-modified samples exhibited three components assigned to bulk, surface, and carbon-bonded oxygen, indicating successful chemical modification. Quantitative evaluation based on the relative areas of the three peaks showed modification ratios below 10% for every temperature/time combination, with no systematic dependence on either parameter. For the 1-hexanol modification, a similar XPS analysis at the O 1s region was performed (Figure 5b). Its modification ratio was estimated to be 8.8%, which was similar to that obtained with the 6AH modifications.

### 3.3. Functional Evaluations of Composite Films

**Figure 8:**
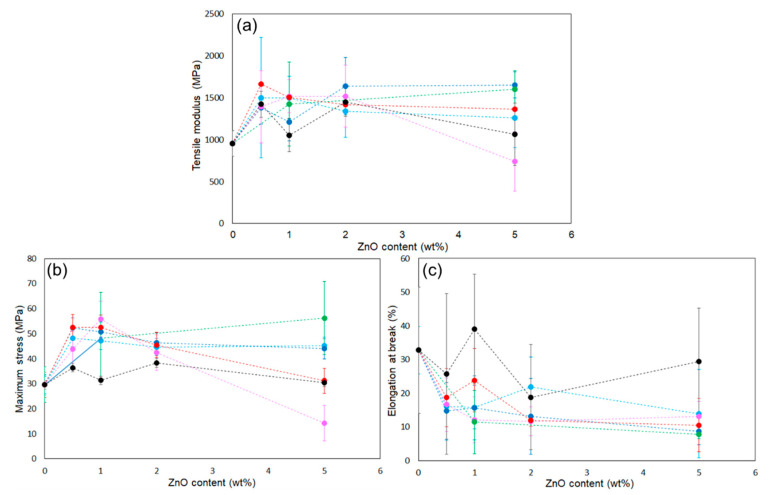
Plots of (**a**) tensile modulus, (**b**) maximum strength, and (**c**) elongation at break of ZnONP-120-1 (black), 6AH-modified ZnONP-80-1 (light blue), 6AH-modified ZnONP-80-24 (navy blue), 6AH-modified ZnONP-120-1 (pink), 6AH-modified ZnONP-120-24 (red), and 1-hexanol-modified ZnONP-120-1 (green).

**Figure 9:**
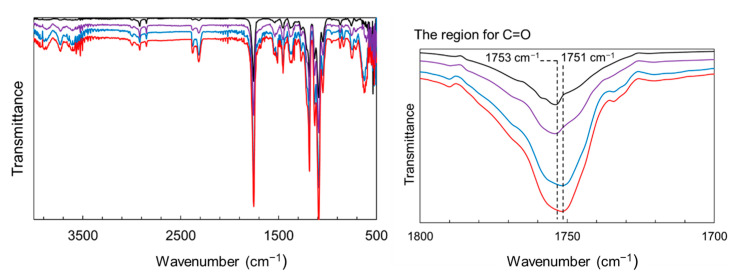
FT-IR spectra of PLA (black line), composite films with unmodified ZnONP (purple line), 1-hexanol-modified ZnONP (blue line), and 6AH-modified ZnONP (red line).

The reinforcing potential of the surface-modified particles was assessed in PLA composite films. Figure S1 shows the appearance of the films: the neat PLA was uniform and transparent, whereas the ZnONP-containing films were opaque. In addition, Figure S4 presents the transmittance spectra of the films over the wavelength range of 200–800 nm. The composite film exhibited lower transmittance than the neat PLA film over the visible light range. The transmittance at 400 nm was detected to be 95.8%, 54.5%, and 37.1% for neat PLA, 6AH-ZnONP(1 wt%)/PLA, and 6AH-ZnONP(5 wt%)/PLA films, respectively, indicating that incorporation of ZnONPs induced Mie scattering due to the increased light scattering from the particles with sizes comparable to the wavelengths of visible light. Mechanical properties were measured by tensile testing. Representative stress–strain curves are displayed in Figure S5, and elastic modulus, maximum stress, and elongation at break are plotted against ZnONP content in Figure 8. The incorporation of ZnONPs, whether functionalized or not, generally increased the tensile modulus and reduced elongation at break relative to neat PLA, indicating reduced deformability. Regarding maximum stress, functionalized ZnONP provided higher strength than unmodified ZnONP at loadings ≤ 2 wt%. The maximum strength ratios of the composite films (1 wt%) to neat PLA film, *σ*_ZnONP_/*σ*_PLA_, *σ*_hexanol-ZnONP_/*σ*_PLA_, and *σ*_6AH-ZnONP_/*σ*_PLA_, were calculated to be 1.06. 1.63, and 1.89, respectively. The introduction of the amino group on the ZnONP surface significantly improved the composite film strength. Although no clear correlation emerged between the XPS-determined functionalization ratio (Table 1) and film strength, the mechanical trends were consistent across all synthesis conditions without 5 wt% containing films. To investigate the effect of hydrogen bonding [48], FT-IR measurements were performed (Figure 9). In the case of PLA and the composite with unmodified ZnONP, peaks corresponding to a stretching vibration of a carbonyl unit were detected at 1753 cm^−1^, whereas, for the composite with modified ZnONP, these were low-wavenumber shifts to 1751 cm^−1^. These results suggest hydrogen bonding between the carbonyl unit of PLA and the functional group of ZnONP, although the shift is not clear, probably due to the small probability of ZnONPs near the surface. The intermolecular interactions between the functional groups and the polymer matrix likely affected the mechanical performance. In the film containing 5 wt%, large variations were observed, and the average values differed significantly depending on the synthetic conditions.

**Figure 10:**
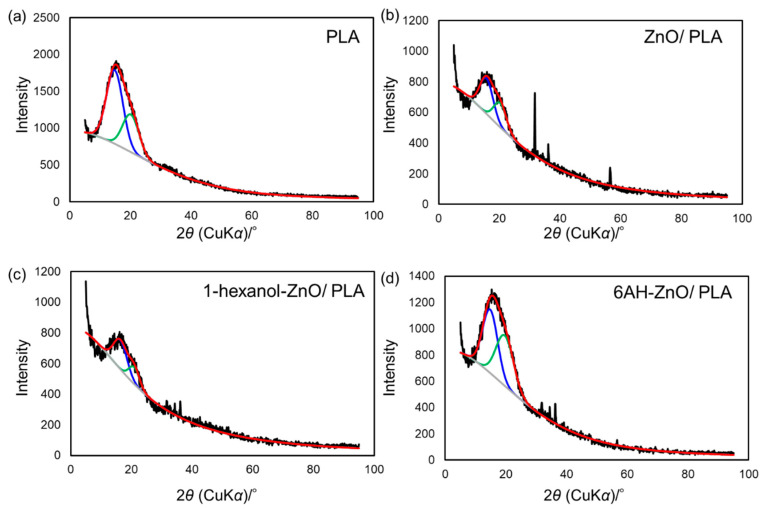
XRD patterns of PLA (**a**), unmodified ZnONP/PLA (**b**), 1-hexanol-modified ZnONP/PLA (**c**), 6AH-modified ZnONP/PLA (**d**). Black, red, blue, green, gray lines show experimental data, fitting curve, two diffraction components, baseline fitting, respectively.

**Figure 11:**
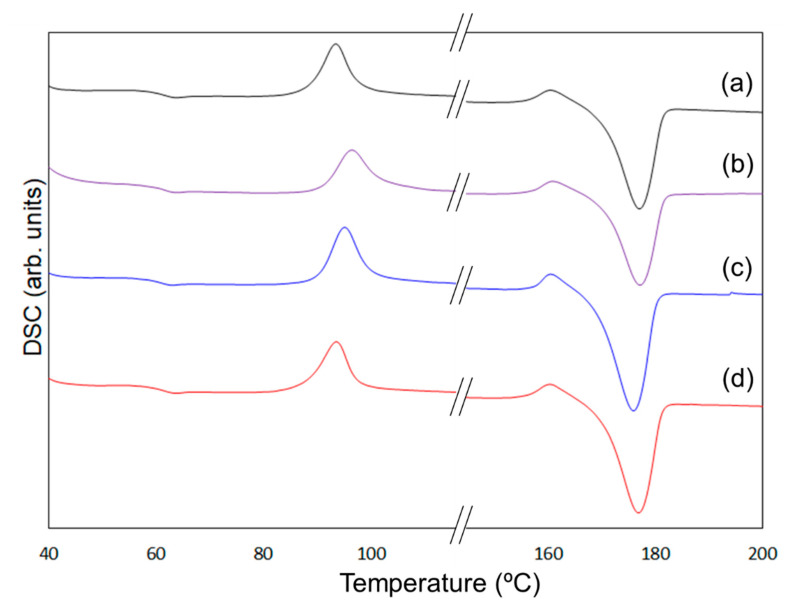
DSC curves of PLA (a), unmodified ZnONP/PLA (b), 1-hexanol-modified ZnONP/PLA (c), 6AH-modified ZnONP/PLA (d).

To clarify the mechanism behind the enhancement of the mechanical properties of the composites containing 6AH-modified ZnONP, despite the surface modification ratio of less than 10%, XRD and DSC analyses were conducted (Figure 10 and Figure 11). For XRD patterns, based on curve fitting by the mixed Gaussian–Lorentzian function, two diffraction components were detected at approximately 15° and 20° in 2*θ*, corresponding to the (203) and (015) lattice plane, respectively [49]. An average value of 2*θ* and area ratios of diffractions are summarized in Table 2. The area ratio assigned to the (015) lattice plane was 48.4% for 6AH-ZnONP, whereas it remained below 40% for all other ZnONPs. This suggests that functionalization affected the crystal structure of PLA, probably due to interactions between the filler and matrix. In addition, the glass transition temperature (*T*_g_), cold crystallization temperature (*T*_cc_), and melt temperature (*T*_m_) were determined by DSC measurements, as summarized in Table 3. The values of the *T*_g_ and *T*_m_ of composites were comparable to those of neat PLA, indicating that the addition of ZnONPs would have no significant effect on the free volume or the energetic stability of PLA crystals. On the other hand, while *T*_cc_ of unmodified ZnONP/PLA increased compared to that of neat PLA, *T*_cc_ of 6AH-modified ZnONP/PLA remained comparable to that of neat PLA. This difference is attributed to the phenomenon that, although ZnONPs suppress polymer chain mobility and thereby elevate *T*_cc_, the introduction of the amino group is expected to act as a nucleating agent, facilitating crystallization at a lower temperature. These results indicate that, despite the surface modification ratio being below 10%, it significantly affected the crystalline structure of PLA and the particle sizes, thereby demonstrating the potential to control the mechanical properties of the composite material.

**Figure 12:**
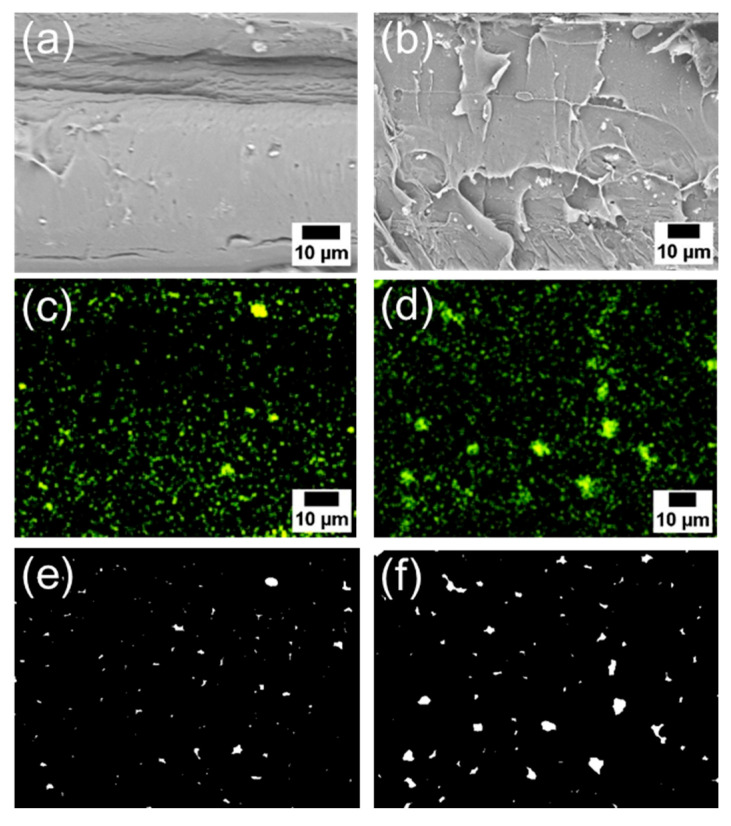
SEM images (**a**,**b**), EDS mappings (**c**,**d**), and binarized images (**e**,**f**) of the cross-sections of the PLA composite films with 6AH-modified ZnONP at 1 wt% (**a**,**c**,**e**) and 5 wt% (**b**,**d**,**f**).

**Figure 13:**
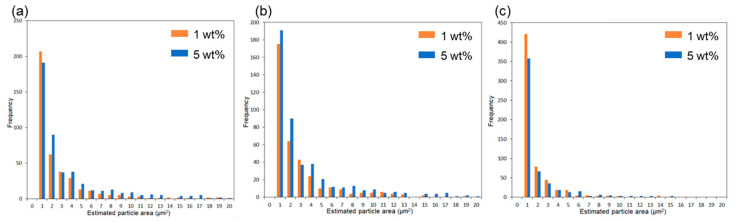
Histograms of particle frequency against estimated particle area from the binarized images of composites containing unmodified (**a**), 1-hexanol-modified (**b**), and 6AH-modified ZnONP (**c**) at 1 and 5 wt%.

Subsequently, the composite using 6AH-ZnONP, which was synthesized at 120 °C for 1 h and exhibited distinct mechanical properties, was subjected to a detailed analysis of its film interior to evaluate the dispersibility of ZnONPs. Figure 12a–d and Figure S6a–h show the SEM images and corresponding EDS mappings of Zn from the cross-sections of the composite films with 6AH-ZnONP and unmodified- and 1-hexanol-ZnONP at 1 and 5 wt% ratios, respectively. The regions where Zn was detected were identified as corresponding to particles. The particle size was evaluated by image analyses based on binarization of the data. Details of the binarization method are provided in the Materials and Methods section. A representative binarized EDS mapping image is shown in Figure 12e,f and Figure S6i–l. Histograms of the particle area were fabricated based on the Zn mapping processed by binarization (Figure 13). The estimated particle sizes and ratios of nanometer-sized particles, that is below 1 μm^2^, for the composites with 1 and 5 wt% of modified ZnONP are summarized in Table 4. The composites containing unmodified and 1-hexanol-modified ZnONPs exhibited larger average particle sizes and a lower proportion of particles below 1 μm^2^, respectively, compared to those containing 6AH-modified ZnONP. These findings suggest that the enhanced compatibility between ZnONPs and PLA is attributed to interactions introduced thorough the surface functionalization. The larger variance observed at 5 wt% contents suggests that, despite the reduction in average particle size, the larger particles probably increased due to aggregation at this concentration.

**Figure 14:**
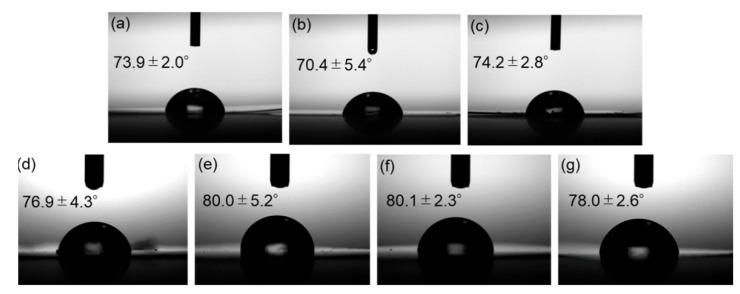
Photographs of neat PLA (**a**) and composite films with unmodified ZnONPs at 1 wt% (**b**) and 5 wt% (**c**); 1-hexanol-modified ZnONPs at 1 wt% (**d**) and 5 wt% (**e**); and 6AH-modified ZnONPs at 1 wt% (**f**) and 5 wt% (**g**). The inserted values indicate contact angles.

Contact angle measurements were carried out to assess the water repellency of the composite surfaces. Side-view images of water droplets on the films and the measured contact angles are shown in Figure 14. Incorporation of surface-modified ZnONP increased the contact angle, indicating reduced surface free energy—presumably as the result of hydrogen bonding between PLA and the functionalized ZnONP.

## 4. Conclusions

In this study, ZnO nanoparticles (ZnONPs) surface-functionalized with an amino group were synthesized via a hydrothermal method, and their potential use as a reinforcing filler in a polylatic acid (PLA) composite film was investigated. Within the range of reaction conditions employed, XPS measurements revealed that the surface modification ratio of the ZnONPs remained below 10%. Composite films with PLA were fabricated and subjected to a tensile test to evaluate their mechanical properties. It was found that films containing 6AH-modified ZnONPs exhibited higher tensile strengths than those with unmodified ZnONPs. Furthermore, an increase in water contact angle indicated that the surface modification enhanced the hydrophobicity of the films. In addition, SEM–EDS analyses of the film cross-sections, combined with image analyses, confirmed nanoparticle aggregation; however, no clear dependence on ZnONP content was observed.

This study demonstrated the potential of surface-functionalized ZnONPs as reinforcing fillers, highlighting a promising approach for the development of next-generation sustainable materials. The methodology established here is expected to serve as a foundational technology for the creation of such materials. In particular, due to its low cost and minimal environmental impact, this approach holds significant promise for a wide range of applications, not only in industrial films but also in medical and food-related materials.

## Figures and Tables

**Table 1:** Binding energy and peak areas for the O 1s region of each XPS analysis. ^a^ The component attributable to carbon-bonded oxygen was too weak to appear as a distinct peak.

	Average Binding Energy (eV)			Peak Area (%)	
	Zn-O	Zn-OH	C-O	ZnO + Zn-OH	C-O
Modified ZnONP-80-1	530.7	531.4	532.5	92.2	7.8
Modified ZnONP-120-1	530.6	531.4	532.8	98.1	1.9
Modified ZnONP-80-24	530.6	531.4	532.7	94.2	5.8
Modified ZnONP-120-24	530.6	531.4	532.7	94.3	5.7
Unmodified ZnONP-120-1	530.7	531.5	n. d. ^a^	-	-

**Table 2:** Estimated average and peak area ratio of diffraction components from XRD patterns.

	Diffraction Component 1		Diffraction Component 2	
	Average 2*θ*/°	Peak Area (%)	Average 2*θ*/°	Peak Area (%)
PLA	14.8	66.2	20.1	33.8
Unmodified ZnONP/PLA	15.7	60.5	20.3	39.5
1-hexanol-ZnONP/PLA	16.3	68.6	20.9	31.4
6AH-ZnONP/PLA	14.8	51.6	19.6	48.4

**Table 3:** Evaluated glass transition temperature (*T*_g_), cold crystallization temperature (*T*_cc_), and melting temperature (*T*_m_) from DSC measurements.

	*T*_g_ (°C)	*T*_cc_ (°C)	*T*_m_ (°C)
PLA	61.2	93.4	177.1
Unmodified ZnONP/PLA	61.2	96.4	177.2
1-hexanol-ZnONP/PLA	60.9	95.1	176.0
6AH-ZnONP/PLA	61.6	93.5	176.9

**Table 4:** Estimated particle size and ratios of particle below 1 μm^2^ based on each binarized image.

		Estimated Particle Size (μm)		Ratios of Particle Below 1 μm^2^ (%)
		Average	Standard Deviation	
Unmodified ZnONP/PLA	1 wt%	2.48	4.11	52
	5 wt%	3.36	4.79	40
1-hexanol-ZnONP/PLA	1 wt%	2.37	3.36	48
	5 wt%	2.96	3.84	41
6AH-ZnONP/PLA	1 wt%	0.54	1.04	69
	5 wt%	0.31	1.24	71

## Data Availability

The original contributions presented in this study are included in the article/Supplementary Materials. Further inquiries can be directed to the corresponding author.

## Supplementary Materials

The following supporting information can be downloaded at https://www.mdpi.com/article/10.3390/ma18132986/s1, Figure S1: Appearance of the composite films; Figure S2: X-ray diffraction of the synthesized samples; Figure S3: SEM images and EDS mappings for modified 1-hexanol-ZnONP; Figure S4: UV–vis transmittance spectra of neat PLA, 6AH-ZnONP (1 wt%)/PLA, and 6AH-ZnONP (5 wt%)/PLA films, Figure S5: Typical stress–strain curves of PLA film and composites film; Table S1: Crystallite size of the synthesized samples, Figure S6: SEM images, EDS mappings, and binarized images of the cross-sections of the PLA composite films with unmodified ZnONP and 1-hexanol-modified ZnONP.
