# Supplementary material for: Preparation of Particle-Reinforced Resin Using Highly Functional ZnO Particle Filler Driven by Supramolecular Interactions
Source: Materials (Basel). 2025 Jun 24;18(13):2986. doi: 10.3390/ma18132986 (PMC12251213; doi:10.3390/ma18132986)
Supplement: Supplementary file 1 [file materials-18-02986-s001.zip › materials-3699989-supplementary.pdf]

Supplementary Materials:

1. Additional data for film preparations
2. Additional data for ZnONPs
3. Evaluation of surface modification
4. Additional data for the composite films
References

1. Additional data for film preparations

Figure S1 shows the appearance of the films.

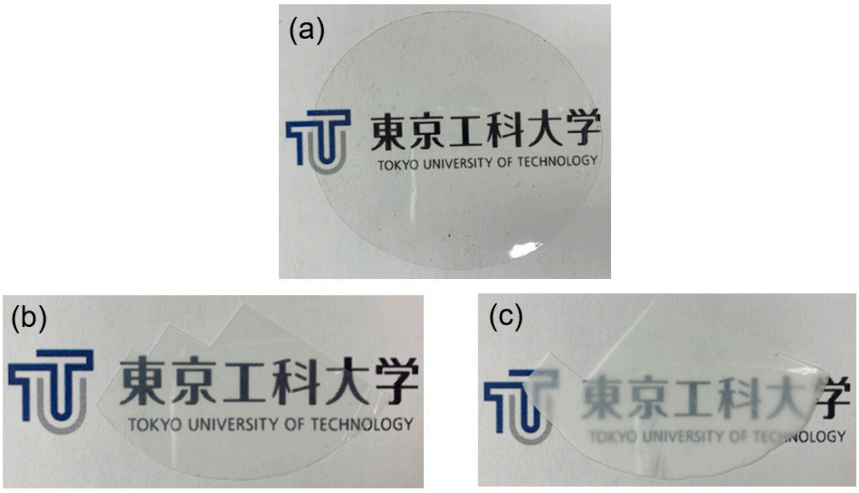

Figure S1. Appearance of the composite films: (a) PLA film, (b) 1 wt% ZnO PLA film, (c) 5 wt% ZnO PLA film.

2. Additional data for structural analyses of ZnONPs

Figure S2 shows the X-ray diffraction of ZnONP modified by 1-hexanol which was synthesized by the same method as 6AH-modified ZnONPs. It was found that the product was hexagonal wurtzite ZnO [1]. Crystallite sizes of the synthesized ZnONPs were summarized in Table S1.

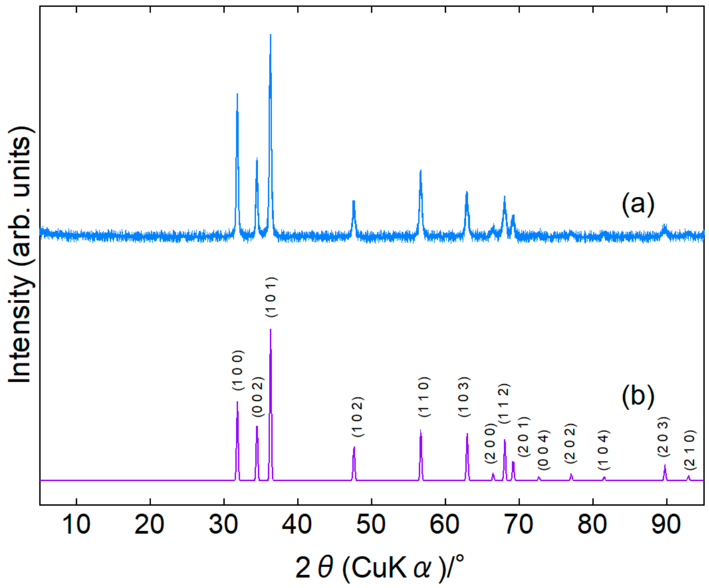

Figure S2. X-ray diffraction of reported data of 1-hexanol-modified ZnONP (a) and wurtzite ZnO (b).

Table S1. Crystallite size of the synthesized samples.

	Crystallite Size (nm)
unmodified ZnONP	24.3 ± 0.8
1-hexanol-modified ZnONP-120-1	25.8 ± 0.8
6AH-modified ZnONP-80-1	24.4 ± 0.9
6AH-modified ZnONP-120-1	31.8 ± 0.6
6AH-modified ZnONP-80-24	24.5 ± 0.7
6AH-modified ZnONP-120-24	24.2 ± 0.4

3. Additional data for evaluation of surface modification of ZnONPs

Figure S3 exhibits SEM image and EDS mappings of ZnO with 1-hexanol, in which it was found that ZnONP was modified with organic spices due to detection of the carbo atom.

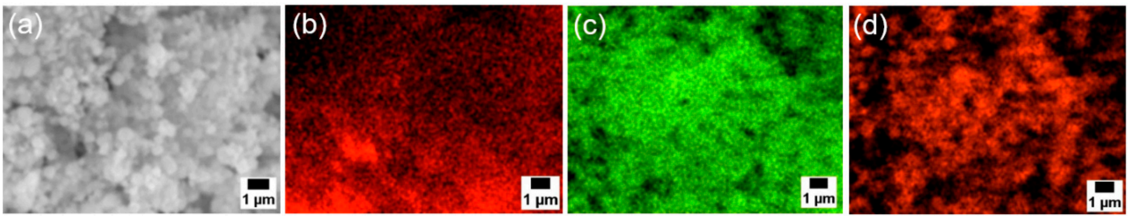

Figure S3. SEM images and (b–d) EDS mappings (b: carbon, c; zinc, d: oxygen) for modified 1-hexanol-ZnONP-120-1.

4. Additional data for the composite films

Figures S4 and S5 indicate UV-vis transition spectra of films, the typical stress-strain curves for each sample, respectively. In addition, SEM images (Figure 6a–d), EDS mappings (Figure 6e–h), and binarized images (Figure 6i–l) of the cross sections of the PLA composite films containing unmodified and 1-hexanol-modified ZnONPs.

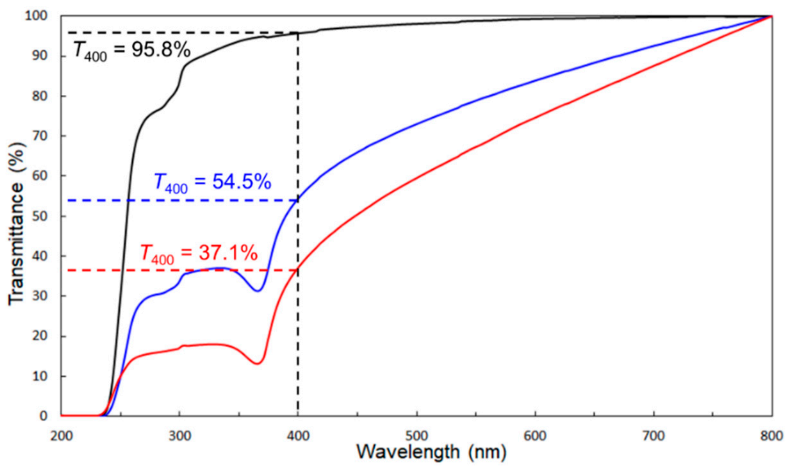

Figure S4. UV–vis transmittance spectra of neat PLA (black line), 6AH-ZnONP (1 wt%)/PLA (blue line), and 6AH-ZnONP(5 wt%)/PLA (red line) films with the transmittance value at 400 nm.

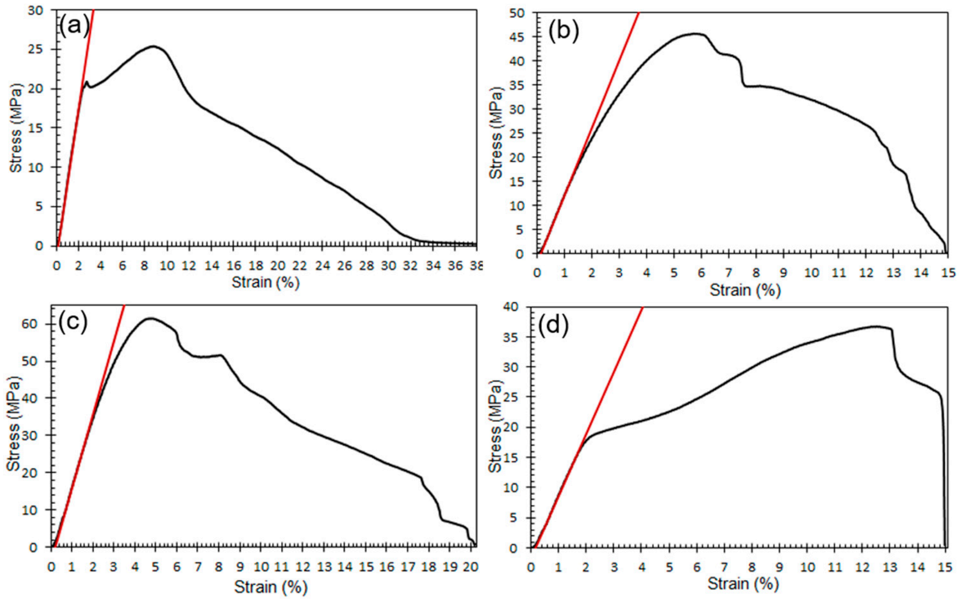

Figure S5. Typical stress-strain curves of PLA film (a), and composites with 1 wt% of unmodified ZnONP 1 wt% (b), with 1 wt% of 1-hexanol modified-ZnONP (c), with 1 wt% of 6AH modified-ZnONP (d).

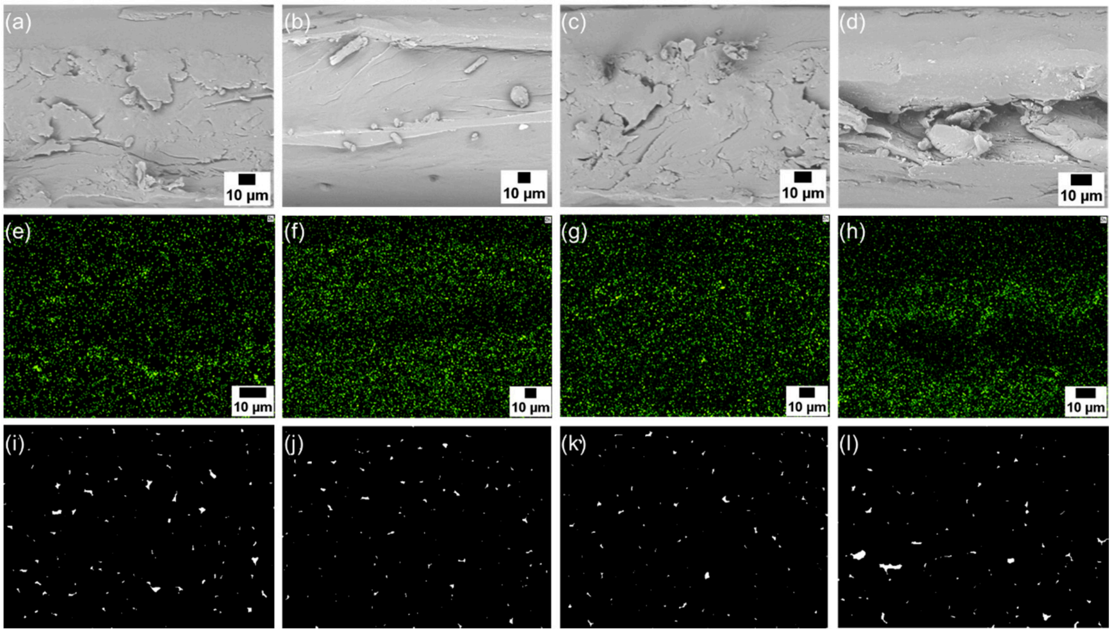

Figure S6. SEM images (a–d), EDS mappings (e–h), and binarized images (i–l) of the cross sections of the PLA composite films with unmodified ZnONP (a, e, i for 1 wt% and b, f, j for 5 wt%) and 1-hexanol-modified ZnONP (c, g, k for 1 wt% and d, h, l for 5 wt%).

References

1. FIZ Karlsruhe—Leibniz Institute for Information Infrastructure. Inorganic Crystal Structure Database (ICSD), Collection Code 26170. Available online: https://icsd.fiz-karlsruhe.de/ (accessed on 25 May 2025).
